# Using Ryff’s scales of psychological well-being in adolescents in mainland China

**DOI:** 10.1186/s40359-018-0231-6

**Published:** 2018-04-20

**Authors:** Jie Gao, Ros McLellan

**Affiliations:** 1 Department of Psychology, Sociology & Politics, Sheffield Hallam University, 42 Collegiate Crescent, Sheffield, S10 2BQ UK; 2 Faculty of Education, University of Cambridge, 184 Hills Road, Cambridge, CB2 8PQ UK

**Keywords:** Adolescents, Psychological well-being, Ryff’s scales, Mainland China

## Abstract

**Background:**

Psychological well-being in adolescence has always been a focus of public attention and academic research. Ryff’s six-factor model of psychological well-being potentially provides a comprehensive theoretical framework for investigating positive functioning of adolescents. However, previous studies reported inconsistent findings of the reliability and validity of Ryff’s Scales of Psychological Well-being (SPWB). The present study aimed to explore whether Ryff’s six-factor model of psychological well-being could be applied in Chinese adolescents.

**Method:**

The Scales of Psychological Well-being (SPWB) were adapted for assessing the psychological well-being of adolescents in mainland China. 772 adolescents (365 boys to 401 girls, 6 missing gender data, mean age = 13.65) completed the adapted 33-item SPWB. The data was used to examine the reliability and construct validity of the adapted SPWB.

**Result:**

Results showed that five of the six sub-scales had acceptable internal consistency of items, except the sub-scale of autonomy. The factorial structure of the SPWB was not as clear-cut as the theoretical framework suggested. Among the models under examination, the six-factor model had better model fit than the hierarchical model and the one-factor model. However, the goodness-of-fit of the six-factor model was hardly acceptable. High factor correlations were identified between the sub-scales of environmental mastery, purpose in life and personal growth.

**Conclusions:**

Findings of the present study echoed a number of previous studies which reported inadequate reliability and validity of Ryff’s scales. Given the evidence, it was suggested that future adolescent studies should seek to develop more age-specific and context-appropriate items for a better operationalisation of Ryff’s theoretical model of psychological well-being.

## Background

Psychological well-being in adolescence has always been a focus of public attention and academic research. Although this concept has been widely researched in adolescent studies, researchers have approached it with different combinations of indicators. To name a few examples, Armsden and Greenberg [1] used self-esteem, life satisfaction and affect status to indicate adolescents’ psychological well-being; Shek [2–5] examined hopelessness, purpose in life and general psychiatric morbidity in addition to life satisfaction and self-esteem in a series of studies about psychological well-being of adolescents. Some other indicators have also been adopted, such as mental health [6], hope [7], anxiety [8, 9] and depression [9]. Apparently, psychological well-being has been used as an umbrella term rather than a theoretical construct in these studies, which poses difficulties in systematically reviewing findings regarding adolescents’ psychological well-being. A lack of systematic approach to investigating psychological well-being may be due to the absence of sound theoretical framework of psychological well-being in adolescent studies. Therefore, it is meaningful to explore whether any established models of psychological well-being can be introduced to adolescent studies.

### Conceptualising adolescent psychological well-being

Well-being is conceptualised in a variety of ways in different fields [10]. In the field of psychology, most researchers agree that well-being indicates optimal psychological functioning and experience in life [11]. Generally speaking, there are two philosophical stances in psychological research on well-being, that is, hedonism which underscores being happy; and eudaimonism which places more emphasis on being meaningful [12]. Different theoretical models of well-being have been proposed in accordance with these two philosophical stances.

Based on hedonism, Diener [13] proposed the construct of subjective well-being (SWB) which refers to an individual’s affective and cognitive evaluations of life. They argued that the feeling of happiness and satisfaction with life is universal, even though what brings about happiness and satisfaction may differ across societies and cultures [14, 15].

On the other hand, eudaimonic theorists argued that it is important for individuals to have a sense of meaning and fulfilment in life [12]. Taking this stance, Ryff [16] proposed a theoretical model of psychological well-being which comprises six different aspects of positive functioning, namely autonomy, environmental mastery, personal growth, purpose in life, positive relations with others and self-acceptance. This model was developed based on a thorough study of human functioning [16]. It has been adopted in a large number of empirical studies conducted in various contexts [17], including three adolescent samples [18–20]. Considering that Ryff’s model was originally developed to reflect adults’ positive functioning [21], existing evidence seems insufficient to substantiate its application to adolescents. Therefore, it is reasonable to further explore if Ryff’s six-factor model can be applied as a sound theoretical framework for investigating adolescents’ psychological well-being. From a theoretical point of view, given that adolescents are going through a transitional period from childhood to adulthood, they are likely to share similar aspects of positive functioning as adults despite putting different weights on each aspect [22]. For example, autonomy is considered as important for both adolescents and adults, but adolescents may express a much stronger need for autonomy than adults [23]. Since Ryff’s model has given a comprehensive account of positive functioning, it is unlikely that it would have failed in reflecting important aspects of adolescents’ psychological well-being. Nevertheless, more empirical evidence is needed to demonstrate whether Ryff’s theoretical model benefits research in adolescents’ psychological well-being.

### Measuring psychological well-being in adolescents

One issue that may potentially prevent researchers from adopting Ryff’s theoretical model is the inconsistent evidence of construct validity of its measurement. Ryff developed the Scales of Psychological Well-being (SPWB) which is composed of six sub-scales in accordance with the six factors of positive functioning, namely autonomy, environmental mastery, personal growth, purpose in life, positive relations with others and self-acceptance [21, 24]. Different versions of SPWB (20-item, 14-item, 9-item and 3-item) have been widely tested with adult samples in a variety of contexts. Yet the construct validity of the SPWB is contentious. While some studies reported relatively sound construct validity of the SPWB [17, 21, 25–27], the others have identified some potential problems, one of which is the uncommonly high correlations between four of the six sub-scales, namely environmental mastery, personal growth, purpose in life and self-acceptance [28–32]. In a recent study of the SPWB, Chen et al. [33] suggested that the factorial structure of the SPWB may vary depending on sample characteristics, such as gender [34], age [27, 35] and cultural background [36]. The three adolescent studies which adopted the SPWB were all conducted in non-western contexts, that is, Iran [18, 19] and Hong Kong [20]. While very little information of the construct validity of the SPWB was reported in the Iran studies, the Hong Kong study provided promising evidence of applying the SPWB to adolescents, despite some previous studies of the SPWB in Chinese contexts reported inconsistent findings of its construct validity [36, 37]. Accordingly, the present study made an attempt to validate the SPWB in a sample of adolescents in mainland China. The aim was twofold: to generate more empirical evidence of applying Ryff’s theoretical model to investigating adolescents’ psychological well-being; and to contribute to the debate of the construct validity of the SPWB in Chinese contexts.

## Method

This study took a two-step approach to evaluate the application of the SPWB in adolescents in mainland China. Firstly, a pilot study was conducted to adapt the items for Chinese adolescents. Secondly, survey research was conducted to examine the reliability and construct validity of the adapted SPWB. Ethical approval of research was granted by the Faculty of Education Standing Panel on Research Ethics, University of Cambridge. Formal written consents were obtained from participants and their parents or legal guardians before participation.

### Pilot study

Cheng and Chan [36] validated a Chinese version of the SPWB, which was drawn on in the present study. Cheng and Chan [36] selected four items for each sub-scale based on a rigorous process of item selection. Yet, the reliability (Cronbach α ranges from .55 to .70) and construct validity (CFI ranges from .79 to .93, SRMR ranges from .058 to .099) seemed to be inadequate according to the commonly accepted criteria (see more details in Result section) [38]. They suggested that further refinement of items was necessary in order to gain a more psychometrically sound measure of PWB for Chinese samples. According to Van Dierendonck [32], sub-scales with 6–8 items seemed to yield better reliability and construct validity of the SPWB. Hence, it was anticipated that the psychometric quality of Cheng and Chan’s [36] measure would be enhanced by adding more items to each subscale. As a result, on the basis of the 4 items chosen by Cheng and Chan [36], two or three more items were selected from Ryff’s scales for each subscale according to the factor loadings in previous studies and the compatibility with the present context. These items were evaluated in focus group discussion with Chinese adolescent participants. Two focus groups with three and four participants in each group were carried out to check the wording of each item as well as the relevance of each item to the daily life of adolescents. Based on the discussion, the wording of some items was modified to improve the ease of use for adolescents.

Subsequently, the refined SPWB was tested in a small-scale pilot study. A total number of 90 Chinese adolescents (57 boys and 33 girls, mean age 14.17 years) completed the SPWB. The data was analysed in reliability tests and Exploratory Factor Analysis. Based on the results of data analysis, seven items that had low internal consistency and low factor loadings were excluded from the final scale. As a result, a final 33-item Chinese version SPWB was produced for further examination (see Appendix for the items).

### Main survey research

#### Participants

Participants were recruited from three junior high schools in a city in East China. The schools are all typical government-funded junior high schools. Invitations for participating in the research were sent out to first-year students (Grade 7, normally aged 12–13 years) in the three schools. Out of the 1073 invitations sent, 772 adolescents (71.9%) returned signed parent consent forms. These adolescents participated in the questionnaire survey. The mean age was 13.65 years (SD = .39), ranging from 12 to 15 years old. The boy to girl ratio was 47.3% to 51.9% (365 boys to 401 girls, 6 missing gender data).

#### Measure

The adapted SPWB measure consists of 33 items. There are six sub-scales corresponding to the six aspects of positive functioning. The sub-scale of Autonomy assesses the sense of self-determination and freedom from norms. It contains five items, for example, “I tend to be influenced by people with strong opinions”. The sub-scale of Environment Mastery assesses the belief of one’s ability to manage life events. It contains six items, for example, “In general, I feel I am in charge of the situation in which I live”. The sub-scale of Personal Growth assesses one’s openness to new experiences and growth. It contains six items, for example, “For me, life has been a continuous process of learning, changing, and growth”. The sub-scale of Purpose in Life assesses the sense of purpose and meaningfulness in life. It contains five items, for example, “I enjoy making plans for the future and working to make them a reality”. The sub-scale of Positive Relations with Others assesses the extent of having satisfying relationships with others. It contains six items, for example, “I often feel lonely because I have few close friends with whom to share my concerns”. The sub-scale of Self-acceptance assesses one’s attitude towards oneself. It contains five items, for example, “For the most part, I am proud of who I am and the life I lead”. Participants were asked to indicate how accurately each item describes themselves by rating on a 5-point Likert scale ranging from “least like me” (1) to “most like me” (5).

#### Procedure

Group survey sessions were scheduled with each participating school. The first author administered all the paper-and-pen survey with adolescents in class. Detailed instructions were given orally before participants started filling in the questionnaire. Participants were debriefed after they completed the questionnaire.

## Results

### Factor structure

Confirmatory Factor Analysis (CFA) was carried out in AMOS 21 to examine the factor structure of the 33-item SPWB. The method of Maximum Likelihood (ML) with bootstrapping was employed. Standard errors, parameters and model test statistics were calculated using bootstrapping.

In accordance with several previous studies [24, 36, 37], three models were examined in the present study, namely, one-factor model, six-factor model and hierarchical model. To be more specific, the one-factor model suggested that all 33 items were unanimously loaded on a single factor. The six-factor model suggested that the items of each sub-scale were loaded on its corresponding factor and the six factors were correlated with each other. The hierarchical model suggested that the items of each sub-scale were loaded on its corresponding factor and the six factors were subsequently loaded on a higher-order factor, which according to Ryff and Keyes [24] represented the construct of PWB.

The model fit indices of each model are illustrated in Table 1. According to Kline [38], the following model fit indices and corresponding criteria were taken into account. Firstly, the model chi-square χ2, which tests the exact-fit hypothesis that the population covariances are the same as the model-predicted covariances, was presented. Smaller chi-square χ2 value is preferred [38]. Secondly, the Benlter Comparative Fit Index (CFI), which measures the relative improvement in the fit of the tested model over that of a baseline model, was presented in combination with Standardized Root Mean Square Residual (SRMR), which illustrates the overall difference between the observed and predicted correlations. According to Hu and Bentler [39], a CFI value above .95 (the greater, the better) together with a SRMR value smaller than .08 (the smaller, the better) indicates acceptable model fit. Meanwhile, the Steiger-Lind root mean square error of approximation (RMSEA) was presented. According to Bowen and Guo [40], RMSEA smaller than .05 indicates good fit; RMSEA ranging from .05 to .08 indicates fair fit; RMSEA ranging from .08 to .10 indicates mediocre fit. Finally, the Akaike Information Criterion (AIC), which estimates the relative model fit to other models, was presented to facilitate model selection. Model with smaller AIC is preferred [38]. Since these model fit indices examine the model fit from different perspectives, it is important to hold a holistic view when scrutinizing model fit based on the aforementioned indices.

**Table 1:** The model indices of the three CFA models

Models	χ^2^	*df*	CFI	SRMR	RMSEA [90% CI]	AIC
One-factor model	2507.64*	495	.74	.065	.073 [.070, .076]	2639.64
Six-factor model	1726.42*	480	.84	.058	.058 [.055, .061]	1888.42
Hierarchical model	1804.38*	489	.83	.059	.059 [.056, .062]	1948.39

*χ*^*2*^ chi-square, *df* Degree of freedom, *CFI* Comparative Fit Index, *SRMR* Standardized Root Mean Square Residual, *RMSEA* Standardized Root Mean Square Residual, *AIC* Akaike Information Criterion, * *p* <.05

Accordingly, the indices suggested that the one-factor model had very poor model fit. This rejected the hypothesis that all 33 items constitute a unified construct without distinction. In contrast, the six-factor model had the best model fit among the three models, albeit still not good enough according to the aforementioned criteria. Nonetheless, all the items were loaded on the corresponding sub-scale with a factor loading ≥ .30 (see Table 2 for the factor loadings of items). The six sub-scales were closely correlated with each other. Table 4 shows the correlations between the sub-scales in the six-factor model. It is worth noting that the sub-scales of environmental mastery (EM), purpose in life (PL) and personal growth (PG) had very high correlations (*r* > .80, See Table 3).

**Table 2:** Item factor loadings on corresponding factor in the six-factor model

Items	AU	EM	PG	PL	PR	SA
Item 1	.60	.63	.41	.66	.66	.48
Item 2	.37	.46	.42	.61	.61	.61
Item 3	.42	.66	.70	.70	.58	.72
Item 4	.51	.47	.50	.71	.59	.72
Item 5	.42	.62	.61	.58	.64	.59
Item 6	–	.33	.65	–	.63	–

The item NO. in the first column represents the item order in each sub-scale*; AU* Autonomy, *EM* Environment mastery, *PG* Personal growth, *PL* Purpose in life, *PR* Positive relations with others, *SA* Self-acceptance

**Table 3:** Correlations between the sub-scales in the six-factor model

Sub-scales	AU	EM	PG	PL	PR	SA
AU	–	.58, .78	.52, .74	.61, .81	.43, .63	.53, .75
EM	.68	–	.73, .87	.87, .97	.55, .71	.65, .80
PG	.63	.81	–	.82, .93	.50, .69	.73, .86
PL	.71	.92	.88	–	.51, .68	.63, .78
PR	.53	.63	.60	.60	–	.62, .77
SA	.64	.73	.79	.70	.70	–

The estimates below diagonal are correlation coefficients; the estimates above diagonal indicate the bias-corrected 95% CI of the correlation coefficients with the two estimates as lower and upper bound respectively; *AU* Autonomy, *EM* Environment mastery, *PG* Personal growth, *PL* Purpose in life, *PR* Positive relations with others, *SA* Self-acceptance

Rather than having six correlated factors, the hierarchical model hypothesised that the six sub-scales were first-order factors which clustered on a second-order factor (i.e., psychological well-being). Table 4 illustrates the parameter estimates between the second-order factor (i.e., psychological well-being) and the first-order factors (i.e., the six sub-scales). The parameter estimates ranged from .69 (Positive Relations with others) to .95 (Purpose in Life). Yet, the model fit indices suggested that the hierarchical model had slightly poorer model fit than the six-factor model.

**Table 4:** The parameter estimates of the hierarchical model

Parameter	Standardized estimate	Bias-corrected 95% CI	*P*	*R* ^*2*^
PWB→ AU	.74	[.64, .82]	.002	.55
PWB→ EM	.93	[.89, .97]	.003	.86
PWB→ PG	.91	[.86, .95]	.003	.83
PWB→ PL	.95	[.90, .98]	.002	.89
PWB→ PR	.69	[.62, .76]	.001	.48
PWB→ SA	.82	[.76, .87]	.002	.67

*PWB* Psychological well-being, *AU* Autonomy, *EM* Environment mastery, *PG* Personal growth, *PL* Purpose in life, *PR* Positive relations with others, *SA* Self-acceptance

### Internal consistency of items

The item internal consistency of the six sub-scales was examined in SPSS 21. Table 5 illustrates the Cronbach α coefficients of the six sub-scales in the present study and some previous studies of the SPWB. As can be seen, the present study demonstrated relatively better item internal consistency among the sub-scales than most of previous studies [20, 24, 32, 36, 37]. Except the Autonomy sub-scale (Cronbach *α* = .60), the other five sub-scales had acceptable internal consistency of items (Cronbach *α* ≥ .70).

**Table 5:** Cronbach α coefficients of the six sub-scales across studies

Sub-scale	AU	EM	PG	PL	PR	SA
Present study	.60	.70	.71	.78	.78	.75
Ryff and Keyes (1995)	.37	.49	.40	.33	.56	.52
Van Dierendonck (2004)	.47	.51	.50	.24	.40	.60
Cheng and Chan (2005)	.55	.63	52	.68	.65	.56
Li (2014)	.60	.75	.74	.73	.71	.75
Chan et al. (2017)	.77	.87	.82	.88	.77	.80

*AU* Autonomy, *EM* Environment mastery, *PG* Personal growth, *PL* Purpose in life, *PR* Positive relations with others, *SA* Self-acceptance

## Discussion

As a first step towards introducing Ryff’s theoretical model of psychological well-being to adolescent studies, the present study made an attempt to adapt the SPWB for adolescents in mainland China. Results of the present study contributed to the existing debate about factor structure and internal consistency of the SPWB across contexts.

The present study examined three models of the SPWB, namely, one-factor model, six-factor model and hierarchical model. The CFA results showed that the six-factor model had slightly better model fit than the hierarchical model. This finding was not in favour of Ryff’s theoretical framework which constructed psychological well-being as a second-order factor which the six sub-scales clustered on. Although several studies in western contexts have found better goodness-of-fit in the hierarchical model [24, 32], studies in Chinese contexts (i.e., Hong Kong and Taiwain) [20, 36, 37] have reported similar findings as the present study. Such inconsistent findings of factor structure have raised the question of cultural variance. Cheng and Chan [36] suggested that people in collectivistic or individualistic societies are likely to value different things in life, which results in the different factor structures of psychological well-being. Yet the exact reason why the hierarchical model is not supported in Chinese samples is not clear. Further studies of psychological well-being in collectivistic societies may shed more light on this issue.

In the six-factor model of the present study, high factor correlations were found between the sub-scales of environmental mastery (EM), purpose in life (PL) and personal growth (PG), which may explain the inadequate model fit. This finding echoed a number of previous studies which also found high factor correlations between the three sub-scales across contexts [20, 28–33]. Given the considerable number of studies that have reported this problem, one may raise the question that whether the high correlations between these sub-scales are due to problematic operationalisation of these constructs in existing scales or because differentiating these aspects of positive functioning might not be meaningful [31]. Self-Determination Theory (SDT) [41] may shed some light on this issue.

According to SDT, people have three basic psychological needs, namely competence, autonomy and relatedness. It is not hard to recognise a certain overlap between Ryff’s six factors of psychological well-being and the three basic needs of SDT [41]. While Ryff’s definitions of autonomy and positive relations with others correspond to the basic needs of autonomy and relatedness, respectively, the definitions of environmental mastery (i.e., how well they were managing their life situations), purpose in life (i.e., the extent to which respondents felt their lives had meaning, purpose and direction) and personal growth (i.e., the extent to which they were making use of their personal talents and potential) seem to have reflected the need of competence which refers to “*feeling effective in one’s ongoing interactions with the social environment and experiencing opportunities to exercise and express one’s capacities*” [41]. This may have partially explained the high correlations between the three sub-scales of Ryff’s model. Future studies may seek to address this question with empirical evidence.

Meanwhile, results of the present study also showed that the sub-scales of the SPWB had acceptable internal consistency (Cronbach *α* ≥ .70), except the autonomy sub-scale (Cronbach α = .60). The relatively low item internal consistency of autonomy sub-scale requires further explanation. Considering the characteristics of this Chinese adolescent sample, the low internal consistency of autonomy items may result from the inconsistent responses due to Chinese adolescents’ internalised conflicts about autonomy. Given that adolescents are going through a transactional period, they are likely to experience conflicts between their increasing need for autonomy and the restrains from adults (e.g., parents and teachers) [42]. Moreover, cross-cultural scholars suggested that such conflicts may be especially prevalent in a collectivistic society, like China, where interdependence and obedience are highly valued [43, 44]. Chinese adolescents are more likely to experience inhibition on autonomy than their counterparts in individualistic societies [45]. Hence, it was not entirely surprising to obtain relatively low internal consistency of the autonomy items among the current sample. Nonetheless, further refinement is needed to make the items more culturally appropriate and more relevant to adolescents.

A very recent study validated a Chinese version of the SPWB with adolescent samples in Hong Kong [20]. In line with the present study, Chan et al. [20] also found that the six-factor model had better model fit than the hierarchical model. Despite the high correlations between some factors (e.g., .89 between PG and PL; .88 between EM and SA), Chan et al. [20] reported better model fit and better internal consistency of items for their scale than both the present study and some previous studies [36, 37]. Their findings can be seen as a promising message for researchers who attempt to apply Ryff’s theoretical framework of psychological well-being in Chinese adolescents. Nevertheless, given the difference between Hong Kong and mainland China, it is necessary to further validate their scale with samples of adolescents in mainland China.

## Conclusions

The purpose of this study was to explore whether Ryff’s six-factor model of psychological well-being could be applied in Chinese adolescents. The Scales of Psychological Well-being (SPWB) were adapted for assessing the psychological well-being of adolescents in mainland China. Based on a rigorous process of item selection, translation and adaptation, the present SPWB for Chinese adolescents has 33 items. The results of reliability tests showed that five of the six sub-scales had acceptable internal consistency, except the sub-scale of autonomy. This finding underscored the need for more research to look into the construct of autonomy and its relationship with psychological well-being in Chinese adolescents. Meanwhile, the results of confirmatory factor analysis echoed a number of previous studies which demonstrated that the factor structure of the SPWB was not as clear-cut as the theoretical framework suggested [25, 32, 33, 36]. In the present study, the six-factor model had slightly better model fit than the hierarchical model, yet high factor correlations were identified between the sub-scales of environmental mastery, purpose in life and personal growth.

Given the existing evidence, it was sensible to reach the following conclusions. Rooted in eudemonism, Ryff’s six-factor model of psychological well-being has the advantage of comprehensively encompassing different aspects of positive functioning. It provides a promising theoretical framework to investigate psychological well-being. However, the application of this model has suffered from lacking consistent evidence of psychometric quality of its measures across contexts. Hence, in attempts to adopt this theoretical framework to inform investigation of adolescents’ psychological well-being, future studies may seek to develop more age-specific and context-appropriate items of the six factors of psychological well-being. In this way, better operationalisation of this theoretical model can be gained, which will benefit future investigation into adolescents’ psychological well-being.

## Appendix

**Table 6:** The items of the SPWB. The selected items of the adapted SPWB of present study

AU1	I have confidence in my opinions, even if they are contrary to the general consensus.
AU2	I tend to be influenced by people with strong opinions.
AU3	Sometimes I change the way I act or think to be more like those around me.
AU4	My decisions are not usually influenced by what everyone else is doing.
AU5	I often change my mind about decisions if my friends or family disagree.
EM1	I am good at juggling my time so that I can fit everything in that needs to get done.
EM2	The demands of everyday life often get me down.
EM3	In general, I feel I am in charge of the situation in which I live.
EM4	I have difficulty arranging my life in a way that is satisfying to me.
EM5	I am quite good at managing the many responsibilities of my daily life.
EM6	I feel I do not have enough time everyday.
PG1	I am the kind of person who likes to give new things a try.
PG2	I enjoy seeing how my views have changed and matured over the years.
PG3	I have the sense that I have developed a lot as a person over time.
PG4	When I think about it, I haven’t really improved much as a person over the years.
PG5	I think it is important to have new experiences that challenge how you think about yourself and the world.
PG6	For me, life has been a continuous process of learning, changing, and growth.
PL1	I am an active person in carrying out the plans I set for myself.
PL2	I don’t have a good sense of what it is I’m trying to accomplish in life.
PL3	Some people wander aimlessly through life, but I am not one of them.
PL4	I enjoy making plans for the future and working to make them a reality.
PL5	When I think about the future, I feel hopeful.
PR1	I often feel lonely because I have few close friends with whom to share my concerns.
PR2	I find it difficult to really open up when I talk with others.
PR3	It seems to me that most other people have more friends than I do.
PR4	I know that I can trust my friends, and they know they can trust me.
PR5	I don’t have many people who want to listen when I need to talk.
PR6	I have not experienced many warm and trusting relationships with others.
SA1	When I compare myself to friends and acquaintances, it makes me feel good about who I am.
SA2	I like most aspects of my personality.
SA3	In general, I feel confident and positive about myself.
SA4	For the most part, I am proud of who I am and the life I lead.
SA5	Everyone has their weaknesses, but I seem to have more than my share.

## Abbreviations

AIC: Akaike Information Criterion
AU: Autonomy
CFA: Confirmatory Factor Analysis
CFI: Comparative Fit Index
EM: Environment mastery
ML: Maximum Likelihood
PG: Personal growth
PL: Purpose in life
PR: Positive relations with others
RMSEA: Standardized Root Mean Square Residual
SA: Self-acceptance
SDT: Self-Determination Theory
SPWB: Scales of Psychological Well-being
SRMR: Standardized Root Mean Square Residual
